# Continued Versus Interrupted Oral Anticoagulation During Transcatheter Aortic Valve Replacement in Patients With Atrial Fibrillation: A Meta-Analysis

**DOI:** 10.1097/MJT.0000000000001979

**Published:** 2025-05-21

**Authors:** Hritvik Jain, Nandan Patel, Muhammad Daoud Tariq, Ali Saad Al-Shammari, Rozi Khan, Jyoti Jain, Rahul Patel, Faizan Ahmed, Raheel Ahmed, Thomas Alexander

**Affiliations:** 1 Department of Cardiology, All India Institute of Medical Sciences (AIIMS), Jodhpur, India;; 2 Department of Internal Medicine, Foundation University Medical College, Islamabad, Pakistan;; 3 Department of Medicine, College of Medicine, University of Baghdad, Baghdad, Iraq;; 4 Department of Internal Medicine, University of Pittsburgh Medical Center, Harrisburg, PA;; 5 Department of Internal Medicine, University of North Carolina Health Blue Ridge, Morganton, NC;; 6 Department of Cardiology, Duke University Hospital, Durham, NC;; 7 Department of Cardiology, National Heart and Lung Institute, Imperial College London, London, United Kingdom; and; 8 Department of Cardiology, Corpus Christi Medical Center, Corpus Christi, TX.

**Keywords:** anticoagulation, transcatheter aortic valve replacement, aortic stenosis, TAVR

## Abstract

**Introduction:**

A substantial number of patients undergoing transcatheter aortic valve replacement (TAVR) require long-term oral anticoagulants (OAC) owing to comorbidities. This study examined whether continuing oral anticoagulation periprocedurally during TAVR is as safe and effective as interrupting it.

**Methods:**

A systematic search of the major databases was performed to identify relevant studies. Effect estimates were calculated using risk ratios (RR) and 95% CIs by pooling the data using the inverse-variance random effects model. Statistical significance was set at *P* < 0.05.

**Results:**

Four studies were included, with 2962 patients undergoing TAVR with continued OAC (n = 1318) and interrupted OAC (n = 1644). The pooled analysis demonstrated that TAVR with continued OAC had comparable risks for all-cause mortality (RR: 0.91; 95% CI, 0.62–1.34; *P* = 0.64), cardiovascular mortality (RR: 0.89; 95% CI, 0.43–1.84; *P* = 0.76), stroke (RR: 0.67; 95% CI, 0.42–1.08; *P* = 0.09), closure device failure (RR: 0.86; 95% CI, 0.47–1.59; *P* = 0.64), major/life-threatening bleeding (RR: 0.93; 95% CI, 0.74–1.15; *P* = 0.49), and major vascular complications (RR: 0.97; 95% CI, 0.79–1.20; *P* = 0.80) compared with TAVR with interrupted OAC.

**Conclusions:**

In patients undergoing TAVR, continued OAC showed comparable safety and efficacy with interrupted OAC. These findings demonstrate that continuing OAC in the periprocedural period may be a viable option in patients with atrial fibrillation because of comorbidities requiring anticoagulants.

## Abstract 2

Supplemental Digital Content is Available in the Text.

## INTRODUCTION

Atrial fibrillation (AF) is one of the most prevalent arrhythmias worldwide and is characterized by irregular and often rapid heart rhythms due to abnormal electrical activity in the atria.^1^ The prevalence of AF is particularly high in elderly populations and is significantly associated with other cardiovascular conditions such as aortic stenosis (AS). In patients with AS, the increased pressure overload in the left heart chambers due to the narrowed aortic valve increases the risk of developing AF. This co-occurrence further complicates clinical management, because AF itself increases the risk of stroke and systemic embolism.^2^

Transcatheter aortic valve replacement (TAVR) has revolutionized the treatment of AS for the past 2 decades, offering a minimally invasive alternative to surgical valve replacement. Initially reserved for patients ineligible for surgery or those with high surgical risk, TAVR is increasingly being adopted for intermediate-risk and even lower-risk patients.^3^ Although TAVR has been widely used in elderly patients, with rates of pre-existing AF in TAVR cohorts reported to range from 16% to 59%, the management of anticoagulation in these patients remains a complex issue owing to the highly thrombogenic nature of AF.^4,5^

Oral anticoagulants (OAC), including vitamin K antagonists (VKA) and direct oral anticoagulants (DOACs), have been shown to significantly reduce the risk of stroke and systemic embolism in patients with AF. Although DOACs offer a more favorable safety profile and improved survival than VKAs, whether to interrupt or continue OAC therapy during TAVR remains uncertain.^6^ Current guidelines suggest interrupting OAC in patients undergoing procedures with a high risk of bleeding; however, this recommendation is not specific to TAVR and is based on general perioperative principles.^7^

Notably, in the context of catheter ablation for AF, uninterrupted OAC has been associated with reduced bleeding complications and similar or lower rates of thromboembolic events than interrupted regimens. Furthermore, the use of uninterrupted DOACs, particularly dabigatran, has shown even more significant reductions in bleeding than VKAs. Given these findings in other procedural settings, evaluating whether continued OAC offers similar benefits in TAVR is crucial, where both thrombotic and bleeding risks are substantial.^8–10^

This systematic review and meta-analysis aimed to address this gap by evaluating the efficacy and safety of continued versus interrupted OAC in patients with AF undergoing TAVR to provide more robust evidence to inform clinical decision-making by including the latest published randomized controlled trials and observational studies.

## MATERIALS AND METHODS

This meta-analysis was conducted following the recommendations of the Cochrane Collaboration and adhered to the PRISMA 2020 guidelines for systematic reviews and meta-analyses (see **Table 1**, **Supplemental Digital Content 1**, http://links.lww.com/AJT/A221).^11^ The study was registered in the PROSPERO International Prospective Register of Systematic Reviews (CRD42024596707).

### Data sources and search strategy

We performed an extensive electronic search using PubMed, EMBASE, Google Scholar, SCOPUS, and Web of Science databases from their inception to October 2024. Our goal was to identify studies assessing the safety and effectiveness of continuous versus interrupted oral anticoagulants (OAC) in patients with atrial fibrillation (AF) undergoing transcatheter aortic valve replacement (TAVR). There were no limitations on the language or publication date. The search strategy used a combination of Medical Subject Headings (MeSH) and free-text terms such as “Continuation,” “Interruption,” “Oral Anticoagulants,” “OAC,” “Vitamin K anticoagulants,” “VKA,” “Direct oral anticoagulants,” “DOAC,” “Transcatheter aortic valve replacement,” “TAVR,” “Transcatheter Aortic Valve Implantation,” “TAVI,” and “Atrial fibrillation.” Boolean operators such as “AND” and “OR” were used in combination with the keywords. We also manually checked the reference lists of relevant articles to ensure comprehensive coverage. The details of the search strategy are provided in the supplementary file (see **Table 2**, **Supplemental Digital Content 1**, http://links.lww.com/AJT/A221).

### Eligibility criteria

#### Inclusion criteria

Using the PICOs framework, the population included patients with AF undergoing TAVR, with the intervention being continued OAC and the control being interrupted OAC. The outcomes considered were all-cause and cardiovascular mortality, stroke, device closure failure, major bleeding, and significant vascular complications.

#### Exclusion criteria

We excluded studies not reporting these outcomes, those involving non-AF, non-peer-reviewed publications, case reports, case series, review articles, editorials, commentaries, and meta-analyses. Studies in languages other than English were also excluded.

### Study selection

All articles from the database search were imported into the EndNote Reference Manager (Version X7.5). Two authors (H.J. and M.D.T.) independently screened the search results and selected the studies that met the inclusion criteria. Full-text articles from potentially relevant studies were included in the final review. Reference lists of previous reviews and meta-analyses were also checked. Duplicate entries were removed, and disagreements were resolved through consensus with a third reviewer (R.A.).

### Data extraction and quality assessment

Two authors (M.D.T. and H.J.) independently extracted the data into a predesigned Microsoft Excel sheet. Discrepancies were resolved by the third author (R.A.). The extracted data included the first author's name, publication year, study design, sample size, baseline characteristics (eg, age, sex, comorbidities), and reported outcomes. Quality was assessed using the modified Cochrane risk-of-bias (RoB 2.0) tool for randomized controlled trials (RCTs) and the Newcastle-Ottawa Scale (NOS) for observational studies.^12,13^

### Outcomes of interest

The primary outcome was all-cause mortality. The secondary outcomes included cardiovascular mortality, stroke, device closure failure, major or life-threatening bleeding events, and significant vascular complications.

### Data synthesis

All statistical analyses were conducted using Cochrane Review Manager (RevMan version 5.4.1) for data synthesis. A random-effects model was used to calculate risk ratios (RR) with 95% confidence intervals (CIs), and a *P*-value of <0.05 was considered statistically significant. Heterogeneity was assessed using Higgins *I*^2^ test, with values of 0%–25% indicating low heterogeneity, 25%–75% moderate heterogeneity, and >75% high heterogeneity.^14^ Leave-one-out sensitivity analysis was used to identify influential studies affecting heterogeneity. Funnel plots were visually inspected for publication bias.

## RESULTS

**FIGURE 1.**
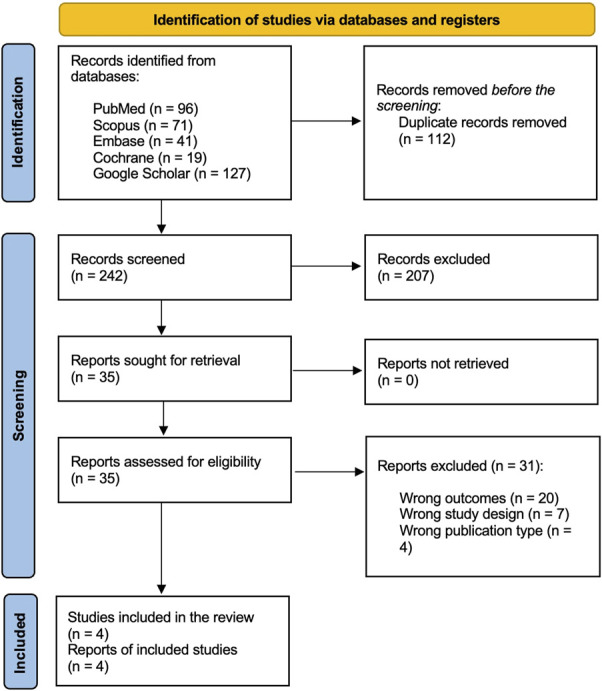
PRISMA flowchart demonstrating the study screening and selection process.

A preliminary database search yielded 354 relevant records. After deduplication (n = 112), 242 records were subjected to primary screening based on title and abstract. A total of 207 records were removed on the primary screening, subsequently, 35 records were subjected to a comprehensive full-text assessment. After this, 31 studies were excluded for various reasons, including wrong outcomes (n = 20), wrong study design (n = 7), and wrong publication type (n = 4). Finally, 4 studies were included in the final analysis.^15–18^ A schematic of the process of screening and study selection is depicted in the PRISMA flowchart (Figure 1).

### Study and patient characteristics

Four studies were included in this meta-analysis, with a total of 2973 patients with AF undergoing TAVR (n = 1322 in the continued OAC group and 1651 in the interrupted OAC group). The detailed baseline characteristics of these studies are presented in Table 1. The mean age of participants in the intervention and control groups was 80.89 ± 5.1 and 80.86 ± 7.83 years, respectively. The inclusion and exclusion criteria followed by all included studies are listed in (see **Table 3**, **Supplemental Digital Content 1**, http://links.lww.com/AJT/A221).

**Table 1.** Baseline characteristics of included studies.

Author name, year	Country	Study design	Patient population	Number of participants		Age; [mean ± SD] or [Median (IQR)] or [Median (range)]		BMI; [mean ± SD] or [Median (IQR)] or [Median (range)]		Males (%)		Hypertension (%)		Diabetes (%)		CHA2DS2-VASc score; [mean ± SD] or [Median (IQR)] or [Median (range)]		Previous stroke (%)		PAVD (%)		CAD (%)		Previous MI (%)	
				C-AC	I-AC	C-AC	I-AC	C-AC	I-AC	C-AC	I-AC	C-AC	I-AC	C-AC	I-AC	C-AC	I-AC	C-AC	I-AC	C-AC	I-AC	C-AC	I-AC	C-AC	I-AC
Brinkert, 2019^15^	Europe	Prospective	High-risk patients requiring anticoagulation undergoing TAVR.	186	185	NR	NR	NR	NR	NR	NR	NR	NR	NR	NR	5.1 ± 1.5	5.1 ± 1.5	26 (4)	45 (24)	NR	NR	NR	NR	NR	NR
Brinkert, 2021^16^	Europe	PSM	Patients requiring long-term anticoagulation undergoing TF-TAVR	584	733	82 (78–85)	82 (78–86)	27.1 (24.0–30.8)	27.1 (24.0–30.8)	297 (50.86)	351 (47.89)	525 (90)	664 (91)	204 (35)	277 (38)	5 (4–6)	5 (4–6)	90 (15)	118 (16)	75 (13)	88 (12)	NR	NR	NR	NR
Mangner, 2019^17^	Germany	Retrospective	Patients with AF prescribed OAC at undergoing TF-TAVI	117	299	80 (77–83)	80 (76–83)	27.8 (24.2–31.2)	27.9 (25.0–32.0)	46 (47.9)	127 (42.5)	116 (99.1)	289 (96.7)	49 (42.2)	162 (54.2)	6 (5–6)	5 (5–6)	22 (18.8)	39 (13.0)	15 (12.8)	33 (11)	55 (47)	131 (43.8)	15 (12.8)	46 (15.4)
POPular PAUSE TAVI, 2024	Netherlands	RCT	Patients receiving oral anticoagulation planning to undergo TAVI	435	434	81 ± 5.6	80.9 ± 6.2	26.5 (24.2–29.7)	26.9 (24.3–30.8)	273 (63.3)	289 (67.7)	339 (78.7)	322 (75.4)	128 (29.7)	123 (28.8)	4.5 ± 1.4	4.4 ± 1.4	46 (10.6)	58 (13.5)	79 (18.3)	85 (19.9)	79 (18.3)	85 (19.9)	61 (14.2)	75 (17.6)

CAD, coronary artery disease; C, continuous; I, interrupted; IQR, interquartile range; MI: myocardial infarction; NR, not reported; PAVD: peripheral arterial vascular disease; PSM, propensity score matching; TF-TAVI, transfemoral transcatheter aortic valve implantation.

### End points

**FIGURE 2.**
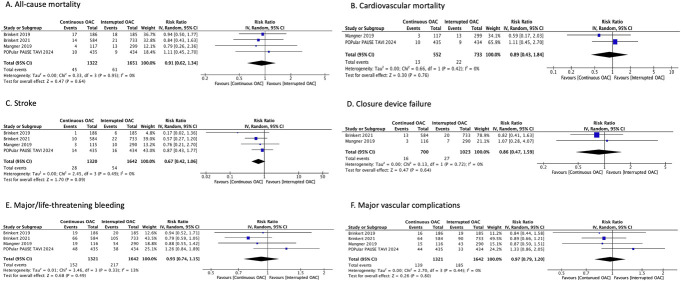
Forest plots for outcomes comparing interrupted and continued oral anticoagulants in patients undergoing TAVR. (A) All-cause mortality, (B) cardiovascular mortality, (C) stroke; (D) closure device failure, (E) major/life-threatening bleeding, (F) major vascular complications.

All four included studies reported all-cause mortality as an outcome.^15–18^ The pooled analysis found that perioperative OAC continuation reduced the risk of all-cause mortality by 9% in comparison with perioperative OAC interruption, but there was no statistical significance between the 2 groups (RR: 0.91; 95% CI, 0.62–1.34; *P* = 0.64; *I*^2^ = 0%) (Figure 2A). No heterogeneity was observed between the studies. Two studies documented outcomes of cardiovascular-related mortality.^17,18^ Our analysis reported that perioperative OAC continuation reduced cardiovascular-related mortality by 11% compared with perioperative OAC interruption (RR: 0.89; 95% CI, 0.43–1.84; *P* = 0.76; *I*^2^ = 0%; Figure 2B). No heterogeneity was observed between the studies.

The outcome of stroke was reported by all 4 studies.^15–18^ Our analysis found that perioperative OAC continuation decreased the risk of stroke by 33% compared with perioperative OAC interruption; however, the results were not statistically significant (RR: 0.67; 95% CI, 0.42–1.06; *P* = 0.09; *I*^2^ = 0%) (Figure 2C). Zero heterogeneity was reported among the studies. Two studies reported data on closure device failure.^16,18^ Our analysis revealed that perioperative OAC continuation decreased the events of closed device failure by 14% compared with perioperative OAC interruption, but the results were not statistically significant (RR: 0.86; 95% CI, 0.47–1.59; *P* = 0.64; *I*^2^ = 0%; Figure 2D). No heterogeneity was observed between the studies.

Major/life-threatening bleeding as an outcome was included in 4 studies.^15–18^ Our analysis revealed that perioperative OAC continuation decreased the major/life-threatening bleeding risk by 7% compared with perioperative OAC interruption; however, the results were not statistically significant (RR: 0.93; 95% CI, 0.74–1.15; *P* = 0.49; *I*^2^ = 13%; Figure 2E). Low heterogeneity was observed among studies. Four studies reported data on major vascular complications.^15–18^ Our pooled analysis showed that perioperative OAC continuation decreased the risk of major vascular complications by 3% compared with perioperative OAC interruption, but there was no statistically significant difference between the 2 groups (RR: 0.97; 95% CI, 0.79–1.20; *P* = 0.80; *I*^2^ = 0%; Figure 2F). No heterogeneity was observed between the studies.

### Risk of bias and publication bias assessment

The risk of bias was “low” in most included studies (see **Table 4 and Figure 1**, **Supplemental Digital Content 1**, http://links.lww.com/AJT/A221). On visual inspection of funnel plots, a symmetrical appearance demonstrated no to low risk of publication bias (see **Figure 2–7**, **Supplemental Digital Content 1**, http://links.lww.com/AJT/A221).

## DISCUSSION

This meta-analysis attempted to address the ongoing debate among cardiologists over the best anticoagulation strategy for patients with comorbidities requiring long-term anticoagulation. This is particularly significant and necessary, because approximately one-third of all TAVR cases include patients with comorbidities necessitating long-term OAC, with most of these patients being affected by AF.^19^ Although the amount and quality of evidence supporting either strategy remain limited, most institutions, in practice, prefer using the interrupted approach as the standard of care, aligning closely with the current international guidelines that broadly recommend the interruption of OAC for patients with comorbidities undergoing a minimally invasive procedure with heightened bleeding risk. These recommendations typically suggest stoppage of DOACs, 24–28 hours, and warfarin 3–5 days before the procedure to mitigate the peri- and postprocedural bleeding risk.^20^ However, these recommendations provide nonspecific guidelines for patients undergoing TAVR, specifically for patients with indications for long-term OAC.^21^ The interplay between anticoagulation strategies and long-term safety outcomes further becomes intricate owing to the heterogeneity of the comorbidities. Moreover, the decision-making process regarding OAC management involves a thorough assessment of individual thromboembolic risk factors, such as CHA_2_DS_2_-VASc scores, alongside bleeding risk assessments, which adds to the complexity of the process.^22^ This warrants a comprehensive and systematic synthesis of evidence to determine the optimal and tailored approach while assessing its safety and effectiveness specifically for this patient population.

Most published literature in this domain is based on retrospective, nonrandomized cohorts, apart from a new noninferiority RCT. To our knowledge, this is the most comprehensive review of this subject to date. Here, we evaluated 6 outcomes comparing the two major strategies for OAC in patients with comorbidities, such as AF, undergoing TAVR. Our analysis included 2973 patients across 4 studies, with 1322 patients and 1651 patients in the continued and interrupted OAC arms, respectively. The patient population was characteristically elderly, hypertensive, and had higher BMI and additional comorbidities. The continued approach demonstrated comparable safety and efficacy with the interrupted approach in all-cause mortality, cardiovascular mortality, stroke, major bleeding, left atrial appendage occlusion device failure rate, and incidence of significant vascular complications. Overall, the continued use of OAC in such patients during the peri- and postinterventional periods does not increase the risk of bleeding, thromboembolic events, and other vascular complications. Thus, our study supports a continued approach for OAC in patients requiring long-term anticoagulation therapy.

The results of our analysis for all-cause and cardiovascular mortality are consistent with those of most studies included in our review, which indicate no significant difference between continued and interrupted anticoagulant therapy considering periprocedural and long-term safety end points. This apparent indifference in the observed outcomes may be attributed to various factors. First, there is variability in the patient selection and risk profiles. These differences may lead to variable outcomes and thus obscure results. Second, the type of OAC used plays a critical role; although some studies suggest survival benefits for patients continuing DOACs,^23^ others did not differentiate between OAC types, complicating outcome comparisons.^24^ In addition, effective perioperative management strategies can mitigate bleeding risks associated with continued therapy, further normalizing the safety results between the 2 approaches.^25^ Finally, variability in study design, such as differences between RCTs and observational studies, can lead to variable findings regarding safety and efficacy outcomes, leading to diminished pooled differences. Overall, although our review suggests no significant differences in mortality outcomes between continued and interrupted therapies, these gaps in the available literature highlight the need for further randomized trials.

Vascular complications remain a notable concern during TAVR procedures, further complicating the outcomes and leading to serious adverse events postprocedure. The current state of the literature reflects that vascular complications occur in approximately 10%–20% of patients undergoing TAVR, with major vascular complications occurring in approximately 5.2%–16.2% in specific patient cohorts.^26^ Another trial documented the occurrence of major vascular complications in approximately 15.3% of patients undergoing TAVR, highlighting the clinical relevance of this issue.^27^ Our study found no significant differences in the risk of vascular complications between the 2 approaches. This is probably because of a better understanding of the mechanisms of bleeding and more comprehensively structured management protocols in place.^25^ Procoagulants such as protamine are now routinely administered at the end of the procedure, lowering the risk of bleeding in patients on anticoagulants.^28^ In addition, operators use ultrasound-guided femoral access, meticulous sheath positioning, and optimized procedural techniques to minimize trauma, which may explain why the bleeding risk remains comparable between continued and interrupted OAC strategies.^29^ In addition, recent advancements in puncture and closure techniques and in handling TAVR-related access site challenges have further helped to achieve desirable postprocedural outcomes.^30^ Improvements in vascular closure devices (VCDs) and periprocedural hemostatic strategies, including the routine use of preclosure techniques with suture- or plug-based devices such as ProGlide and MANTA, have significantly reduced bleeding risks and optimized hemostasis during large-bore access procedures.^31^ These advancements are significant in determining the applicability of the continued approach in routine clinical practice. It is important to acknowledge that most of the included studies were observational, where minor bleeding events might have been underreported or managed conservatively without meeting predefined end points for major bleeding. Future RCTs with standardized bleeding definitions and adjudicated event reporting may provide a clearer understanding of the nuanced effects of OAC on bleeding. Further research should stratify patients based on thromboembolic and bleeding risk profiles to determine in whom continued OAC might be safest and most effective.

We acknowledge that the largest RCT in this domain reported a numerically higher bleeding risk with continued OAC. However, it is crucial to note that this difference did not reach statistical significance in major bleeding events [11.1% and 8.9% (risk difference, 2.2 percentage points; 95% CI, −1.8 to 6.3)], suggesting that the observed trend may be influenced by study's limitation and biases rather than a definitive difference in risk. Moreover, although the overall bleeding events were higher in the continued approach, these bleeding complications were predominantly minor bleeding (VARC-3 type 1, similar to BARC 2), such as periprocedural bleeding complications that result in manual compression or application of a pressure bandage after discharge from the catheterization laboratory.^18^ We, therefore, emphasize that this meta-analysis is hypothesis generating, which promotes special consideration that could be given to a selective group of patients.

Moreover, scarce evidence suggests that continued peri- and postprocedural OAC may reduce thromboembolic risks without significantly increasing bleeding complications; however, the lack of randomized controlled trials specifically addressing this question results in uncertain opinions.^32^ However, this is counterintuitive to the results of our analysis of the risk of stroke, which showed an insignificant difference between the 2 OAC approaches. In contrast, some observational studies^15–17^ have found a significantly reduced stroke risk with continued oral anticoagulation therapy. The primary mechanism of stroke during TAVR is cerebrovascular embolism resulting from the displacement of aortic valve debris or atherosclerotic material from the aorta because of catheter manipulation. In addition, fragmented valvular tissue during balloon aortic valvuloplasty or valve implantation may increase the risk of thrombogenic complications.^33^ Interaction of the debris with exposed endothelial tissue factors activates the coagulation cascade, resulting in macro- and microthrombi formation, leading to cerebrovascular events.^33,34^ Similarly, the periprocedural onset of AF is one of the strongest risk factors for stroke in these patients. As observed in certain individual studies, a continued approach might offer an anticoagulant cover during the procedure, thereby lowering the risk of stroke. This approach may be beneficial in patients with high CHA2DS2-VASc scores or a history of stroke because it favors the risk/benefit ratio as compared with general patients undergoing TAVR.^35,36^ However, owing to the limited number of studies available, the pooled effect of this outcome diminishes and demonstrates nonsuperiority to the interrupted approach in our study. Periprocedural new-onset atrial fibrillation is a known risk factor for stroke in patients undergoing TAVR. Patients with persistent or high-burden AF may benefit from continued anticoagulation to reduce embolic risk.^37^ Although these results suggest a potential benefit of the continued approach in stroke prevention, the inconsistency of the findings warrants further research.

One of the assessed outcomes, the failure rate of the left atrial appendage closure device, is of great significance, particularly for patients with AF. This device prevents thromboembolic complications in patients with AF.^38^ Initial anticoagulation reduces the risk of thrombus formation on the device before endothelialization occurs, which generally takes approximately 45 days.^39^ The effect of continued versus interrupted oral anticoagulation approaches on the success or failure rate of implanted devices is questionable and crucial. The results of our analysis indicate that there are no significant differences between the 2 approaches. This is in agreement with the results of the individual studies under consideration and demonstrates that both anticoagulation strategies are equally efficacious and present no differential challenge in determining the success of the LAA closure device success.^16,17^

The quantitative analysis performed in our study revealed a very low heterogeneity among the included studies. This indicates that the effect sizes were consistent across the included studies and reaffirms the robustness of these findings. These findings are likely replicable across similar clinical settings, enhancing their applicability for tailoring clinical decisions according to patient characteristics. Importantly, the consistency of these findings across various studies suggests a potential need to re-evaluate current international recommendations in a similar domain.

Despite these promising results, our study had multiple limitations. These include the lack of randomized studies, because most of the included studies in our analysis were observational, focusing mainly on specific and nondiverse subsets of the population, limiting the generalizability of the findings. In addition, subgroup analysis based on various baseline confounding characteristics of participants could not be conducted because of <10 included studies, which undermines the validity of the subgroup effect estimate. Third, the relatively small sample size limits the analytical power and necessitates further large-scale, multicenter RCTs to validate our findings. Fourth, the reliance on observational and retrospective studies, which did not involve randomization. This lack of randomization may have obscured the true effect of OAC continuation.

Furthermore, it is plausible that patients who continued OAC were carefully selected based on their baseline bleeding risk, favoring those with better hemostatic profiles. Clinicians may have chosen to interrupt OAC in higher-risk patients (eg, those with anemia, prior major bleeding, frailty, or renal impairment), thereby reducing the apparent bleeding risk in the continued OAC group. This selection bias could have masked a true difference in bleeding risk, leading to the observed comparable outcomes between the 2 groups. Therefore, larger well-powered RCTs to definitively assess the effect of OAC continuation on bleeding risk are required.

Another important limitation of our analysis is that some patients included in the POPULAR TAVI trial and the study by Brinkert et al did not have AF as the primary indication for OAC. Although our meta-analysis focused on patients requiring long-term OAC, the inclusion of patients with non-AF introduces potential heterogeneity in the thromboembolic risk and bleeding outcomes. The indications for OAC in these patients may differ, influencing the observed safety and efficacy of continued versus interrupted OAC strategies. Therefore, our findings should be interpreted with caution, particularly when considering non-AF populations. In the Brinkert et al study, the intervention (continuation vs. interruption of anticoagulation) was not standardized. Therefore, potential unreported differences in timing, dosing, and concurrent heparin/antiplatelet therapy may have influenced their results. Hence, an error due to concurrent heparin and selective bridging therapy cannot be completely excluded. Further stratified analyses and dedicated studies are warranted to determine whether the safety and effectiveness of continued OAC differ based on the underlying indication for anticoagulation.

Finally, we would like to acknowledge that our study does not provide definitive evidence to warrant an immediate change in clinical practice guidelines. This is a meta-analysis predominantly based on observational studies and 1 RCT. Our findings do not establish a new standard of care nor directly challenge existing guidelines. However, our results offer valuable insights to inform patient-specific decision making and contribute to the ongoing discussion regarding optimal anticoagulation strategies in patients undergoing TAVR requiring long-term OAC.

## CONCLUSIONS

In patients undergoing TAVR, continued OAC showed comparable safety and efficacy to interrupted OAC. These findings demonstrate that continuing OAC therapy in the periprocedural period may be a viable option in patients with AF because of comorbidities requiring anticoagulation. However, this decision should be tailored to individual patients, considering relevant clinical factors.

## Supplementary Material
