# Supplementary material for: Continued Versus Interrupted Oral Anticoagulation During Transcatheter Aortic Valve Replacement in Patients With Atrial Fibrillation: A Meta-Analysis
Source: Am J Ther. 2025 May 21;33(1):e31–8. doi: 10.1097/MJT.0000000000001979 (PMC12767628; doi:10.1097/MJT.0000000000001979)

**Supplementary Material**

**Continued Versus Interrupted Oral Anticoagulation During Transcatheter Aortic Valve Replacement in Patients with Atrial Fibrillation: A Meta-Analysis**

**SUPPLEMENTARY TABLES**

**Supplementary Table S1**. The Preferred Reporting Items for Systematic Reviews and Meta-Analyses (PRISMA) 2020 Checklist.

| **Section and Topic** | **Item #** | **Checklist item** | **Location where item is reported** |
| --- | --- | --- | --- |
| **TITLE** | | |  |
| Title | 1 | Identify the report as a systematic review. | 1 |
| **ABSTRACT** | | |  |
| Abstract | 2 | See the PRISMA 2020 for Abstracts checklist. | 1 |
| **INTRODUCTION** | | |  |
| Rationale | 3 | Describe the rationale for the review in the context of existing knowledge. | 3 |
| Objectives | 4 | Provide an explicit statement of the objective(s) or question(s) the review addresses. | 3 |
| **METHODS** | | |  |
| Eligibility criteria | 5 | Specify the inclusion and exclusion criteria for the review and how studies were grouped for the syntheses. | 4, 5 |
| Information sources | 6 | Specify all databases, registers, websites, organisations, reference lists and other sources searched or consulted to identify studies. Specify the date when each source was last searched or consulted. | 3, 4 |
| Search strategy | 7 | Present the full search strategies for all databases, registers, and websites, including any filters and limits used. | 3, 4 |
| Selection process | 8 | Specify the methods used to decide whether a study met the inclusion criteria of the review, including how many reviewers screened each record and each report retrieved, whether they worked independently, and if applicable, details of automation tools used in the process. | 4, 5 |
| Data collection process | 9 | Specify the methods used to collect data from reports, including how many reviewers collected data from each report, whether they worked independently, any processes for obtaining or confirming data from study investigators, and if applicable, details of automation tools used in the process. | 4, 5 |
| Data items | 10a | List and define all outcomes for which data were sought. Specify whether all results that were compatible with each outcome domain in each study were sought (e.g. for all measures, time points, analyses), and if not, the methods used to decide which results to collect. | 4, 5 |
|  | 10b | List and define all other variables for which data were sought (e.g. participant and intervention characteristics, funding sources). Describe any assumptions made about any missing or unclear information. | 4, 5 |
| Study risk of bias assessment | 11 | Specify the methods used to assess risk of bias in the included studies, including details of the tool(s) used, how many reviewers assessed each study and whether they worked independently, and if applicable, details of automation tools used in the process. | 6 |
| Effect measures | 12 | Specify for each outcome the effect measure(s) (e.g. risk ratio, mean difference) used in the synthesis or presentation of results. | 6 |
| Synthesis methods | 13a | Describe the processes used to decide which studies were eligible for each synthesis (e.g. tabulating the study intervention characteristics and comparing against the planned groups for each synthesis (item #5)). | 4, 5 |
|  | 13b | Describe any methods required to prepare the data for presentation or synthesis, such as handling of missing summary statistics, or data conversions. | 4, 5 |
|  | 13c | Describe any methods used to tabulate or visually display results of individual studies and syntheses. | 4, 5 |
|  | 13d | Describe any methods used to synthesize results and provide a rationale for the choice(s). If meta-analysis was performed, describe the model(s), method(s) to identify the presence and extent of statistical heterogeneity, and software package(s) used. | 4, 5 |
|  | 13e | Describe any methods used to explore possible causes of heterogeneity among study results (e.g. subgroup analysis, meta-regression). | 4, 5 |
|  | 13f | Describe any sensitivity analyses conducted to assess robustness of the synthesized results. | 4, 5 |
| Reporting bias assessment | 14 | Describe any methods used to assess risk of bias due to missing results in a synthesis (arising from reporting biases). | NR |
| Certainty assessment | 15 | Describe any methods used to assess certainty (or confidence) in the body of evidence for an outcome. | NR |
| **RESULTS** | | |  |
| Study selection | 16a | Describe the results of the search and selection process, from the number of records identified in the search to the number of studies included in the review, ideally using a flow diagram. | 5, 6 |
|  | 16b | Cite studies that might appear to meet the inclusion criteria, but which were excluded, and explain why they were excluded. | 5, 6 |
| Study characteristics | 17 | Cite each included study and present its characteristics. | 5, 6 |
| Risk of bias in studies | 18 | Present assessments of risk of bias for each included study. | 5, 6 |
| Results of individual studies | 19 | For all outcomes, present, for each study: (a) summary statistics for each group (where appropriate) and (b) an effect estimate and its precision (e.g. confidence/credible interval), ideally using structured tables or plots. | 5, 6 |
| Results of syntheses | 20a | For each synthesis, briefly summarise the characteristics and risk of bias among contributing studies. | 5, 6 |
|  | 20b | Present results of all statistical syntheses conducted. If meta-analysis was done, present for each the summary estimate and its precision (e.g. confidence/credible interval) and measures of statistical heterogeneity. If comparing groups, describe the direction of the effect. | 5, 6 |
|  | 20c | Present results of all investigations of possible causes of heterogeneity among study results. | 5, 6 |
|  | 20d | Present results of all sensitivity analyses conducted to assess the robustness of the synthesized results. | 5, 6 |
| Reporting biases | 21 | Present assessments of risk of bias due to missing results (arising from reporting biases) for each synthesis assessed. | NR |
| Certainty of evidence | 22 | Present assessments of certainty (or confidence) in the body of evidence for each outcome assessed. | NR |
| **DISCUSSION** | | |  |
| Discussion | 23a | Provide a general interpretation of the results in the context of other evidence. | 7, 8, 9 |
|  | 23b | Discuss any limitations of the evidence included in the review. | 9, 10 |
|  | 23c | Discuss any limitations of the review processes used. | 9, 10 |
|  | 23d | Discuss implications of the results for practice, policy, and future research. | 10 |
| **OTHER INFORMATION** | | |  |
| Registration and protocol | 24a | Provide registration information for the review, including register name and registration number, or state that the review was not registered. | 3 |
|  | 24b | Indicate where the review protocol can be accessed, or state that a protocol was not prepared. | 3 |
|  | 24c | Describe and explain any amendments to information provided at registration or in the protocol. | 3 |
| Support | 25 | Describe sources of financial or non-financial support for the review, and the role of the funders or sponsors in the review. | Title Page |
| Competing interests | 26 | Declare any competing interests of review authors. | Title Page |
| Availability of data, code and other materials | 27 | Report which of the following are publicly available and where they can be found: template data collection forms; data extracted from included studies; data used for all analyses; analytic code; any other materials used in the review. | Title Page |

**Supplementary Table S2**. Search strategy for all databases.

| **No.** | **Database** | **Search Strategy** | **Number of Articles** |
| --- | --- | --- | --- |
| 1. | PubMed | ((Interruption) OR (Continuation)) AND ((Atrial Fibrillation) OR (AF)) OR ((Oral Anticoagulants) OR (OAC) OR (VKA) OR (DOAC)) AND ((TAVR) OR (TAVI)) | 96 |
| 2. | Embase | ((Interruption) OR (Continuation)) AND ((Atrial Fibrillation) OR (AF)) OR ((Oral Anticoagulants) OR (OAC) OR (VKA) OR (DOAC)) AND ((TAVR) OR (TAVI)) | 41 |
| 3. | Google Scholar | ((Interruption) OR (Continuation)) AND ((Atrial Fibrillation) OR (AF)) OR ((Oral Anticoagulants) OR (OAC) OR (VKA) OR (DOAC)) AND ((TAVR) OR (TAVI)) | 127 |
| 4. | Scopus | ((TITLE-ABS-KEY (continued)) OR (TITLE-ABS-KEY (interrupted))) AND (TITLE-ABS-KEY (oral AND anticoagulants)) AND (TITLE-ABS-KEY (atrial fibrillation)) AND ((TITLE-ABS-KEY (transcatheter aortic valve AND replacement)) OR (TITLE-ABS-KEY (transcatheter aortic valve implantation))) | 71 |
| 5. | Cochrane | (MeSH descriptor: [Oral Anticoagulants] explode all trees) AND ((MeSH descriptor: [Atrial fibrillation] explode all trees) OR (“AF”)) AND ((MeSH descriptor: [Transcatheter Aortic Valve Replacement] explode all trees) OR (MeSH descriptor: [Transcatheter Aortic Valve Implantation] explode all trees)) | 19 |
| TOTAL | | | 354 |

**Supplementary Table S3:** Inclusion and exclusion criteria in each study.

| **Study (Year)** | **Inclusion criteria** | **Exclusion criteria** |
| --- | --- | --- |
| Brinkert, 2019 | The study included consecutive patients who were on oral anticoagulation therapy, either with VKA or DOACs, indicated for a certain specific co-morbidity, and who had undergone TAVR across three European centers from 2015 to 2018. The primary indication for anticoagulation was atrial fibrillation, which was present in 95% of participants. | Patients were excluded if they had contraindications to TAVR or significant comorbidities that would contraindicate the stopping of anticoagulants. Patients with any history that could affect outcomes, such as a high risk of bleeding, prior cardiac surgeries, recent major bleeding events, or any serious prior cardiovascular accidents, were excluded from the study. |
| Brinkert, 2021 | After propensity-score matching, 584 consecutive patients were enrolled, who required long-term oral anticoagulation and had undergone transfemoral TAVR at five high-flow European hospitals from 2011 to 2019.  There were no specific age limits for inclusion. Patients were split into two groups: one with continued therapy with their preferred oral anticoagulant through the procedure, while the other discontinued the therapy, 2-4 days prior to the procedure. All participants had a clear indication of requiring long-term anticoagulation, primarily atrial fibrillation. | Patients excluded from the registry had contraindications to TAVR, significant comorbidities, or conditions that could complicate the procedure or recovery. Specifically, individuals with active bleeding, severe renal impairment, or those requiring additional interventions that could affect the outcomes were omitted. Patients with strong contraindications against discontinuing the anticoagulant therapy were also excluded from the registry. |
| Mangner, 2019 | The study included 598 patients who were i) diagnosed with AF and were on oral anticoagulation therapy at the time of admission and ii) underwent transfemoral TAVI for severe aortic stenosis or bioprosthetic valve failure in the aortic position. Also, the participants must have been receiving OAC continuously from January 2011 to March 2016 to be eligible for the study.  Additionally, patients were classified based on their choice of anticoagulation and its status during the procedure into three groups: i) interrupted VKA, ii) continued VKA, and iii) continued DOACs. The decision to continue or interrupt OAC also depended on other clinical characteristics and concomitant therapeutic procedures. | The exclusion criteria were not explicitly detailed; however, patients with contraindications to TF-TAVI or those with significant comorbidities that could affect outcomes were likely excluded. This would include individuals with severe renal impairment or those who could not safely undergo the procedure due to other health complications. Furthermore, any patients who did not provide informed consent or whose medical records were incomplete were also excluded from the analysis. |
| POPular PAUSE TAVI, 2024 | 869 participants were included, who fulfilled all of the following criteria: i) should be scheduled for transfemoral or trans-subclavian transcatheter aortic valve implantation with a concurrent indication for long-term anticoagulation, ii) must be on active treatment receiving long-term oral anticoagulation at the time of screening, iii) and are willing to provide signed informed consent prior to being enrolled. | People at higher risk for thromboembolism who cannot safely discontinue their oral anticoagulation therapy are excluded. Patients with safety concerns for undergoing TAVI due to other comorbidities were also dropped from the study. These include patients with a prosthetic mechanical heart valve as well as individuals with presentation of an intracardiac thrombus.  Moreover, any individual who had experience with i) venous thromboembolism in the 3 months preceding TAVI or ii) individuals who had a transient ischemic attack or stroke in the 6 months preceding the TAVI. |

**Supplementary Table S4**. Quality assessment using Newcastle Ottawa Scale.

| **Study (Year)** | **Selection** | | | | **Comparability** | **Outcome** | | | **Total Score** |
| --- | --- | --- | --- | --- | --- | --- | --- | --- | --- |
|  | Representativeness of the exposed cohort | Selection of the non-exposed cohort | Ascertainment of exposure | Outcome of interest was not present at the start of the study |  | Assessment of outcome | Duration of follow-up | Adequacy of follow-up |  |
| Brinkert, 2019 | ☆ | ☆ | ☆ | ☆ | ☆ | ☆ |  | ☆ | 8 |
| Brinkert, 2021 | ☆ | ☆ | ☆ | ☆ | ☆☆ | ☆ | ☆ | ☆ | 9 |
| Mangner, 2019 | ☆ | ☆ | ☆ | ☆ | ☆☆ | ☆ | ☆ | ☆ | 9 |

**SUPPLEMENTARY FIGURES**

**Supplementary Figure S1**. Quality assessment for RCT using ROB 2.0


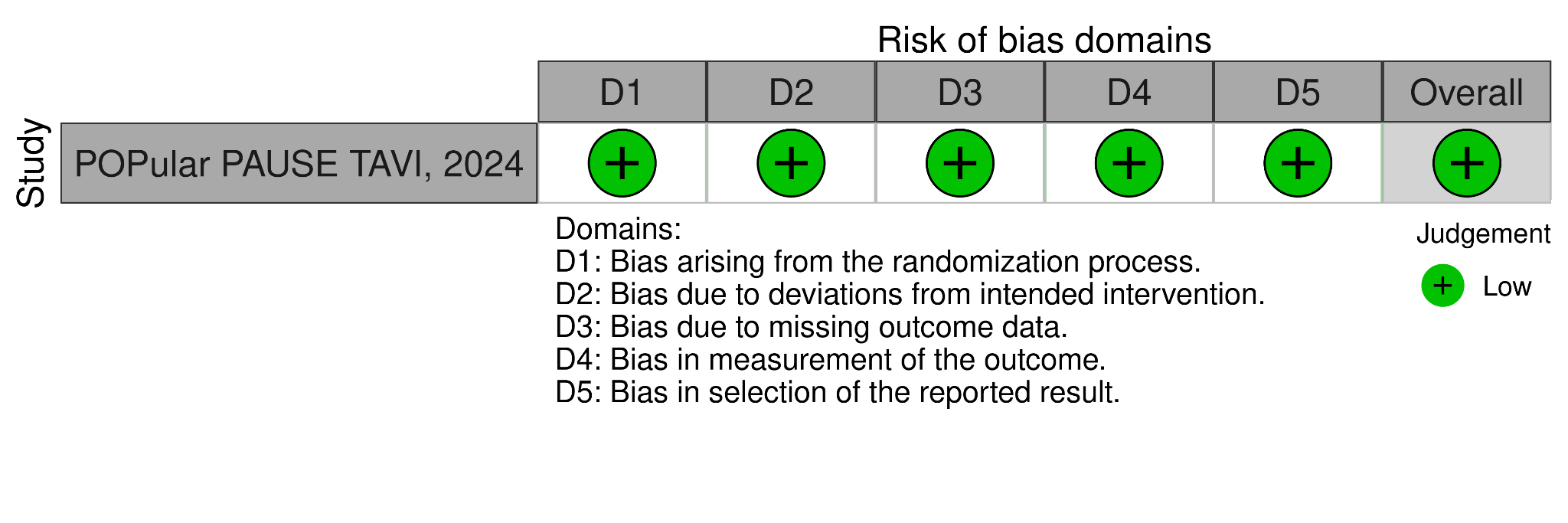


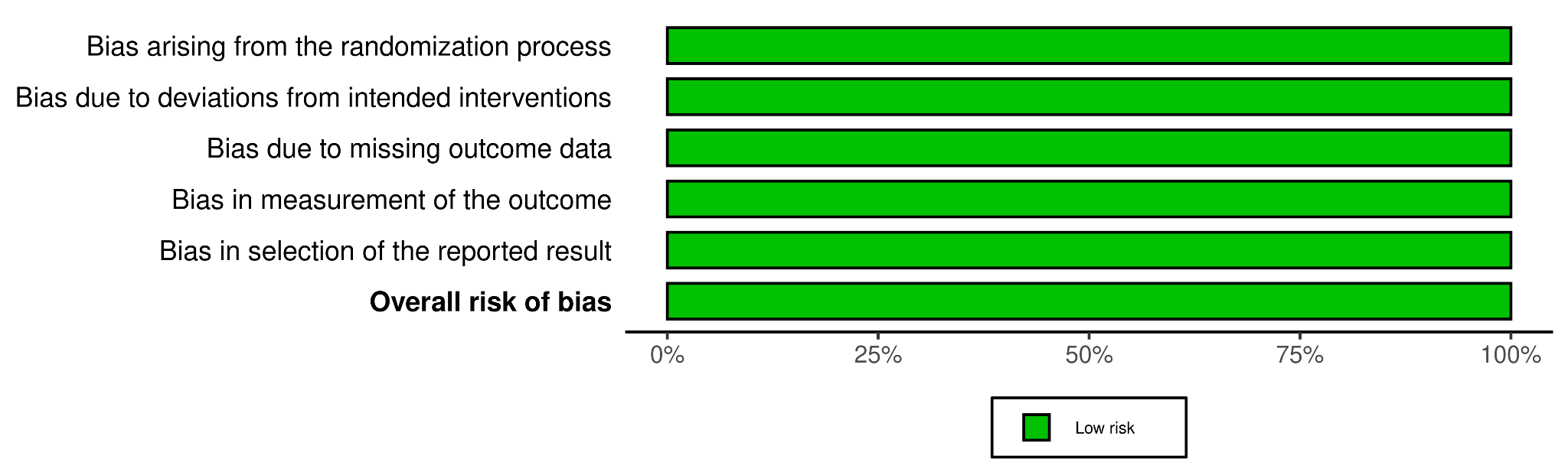


**Supplementary Figure S2**. Funnel plot for all-cause mortality.


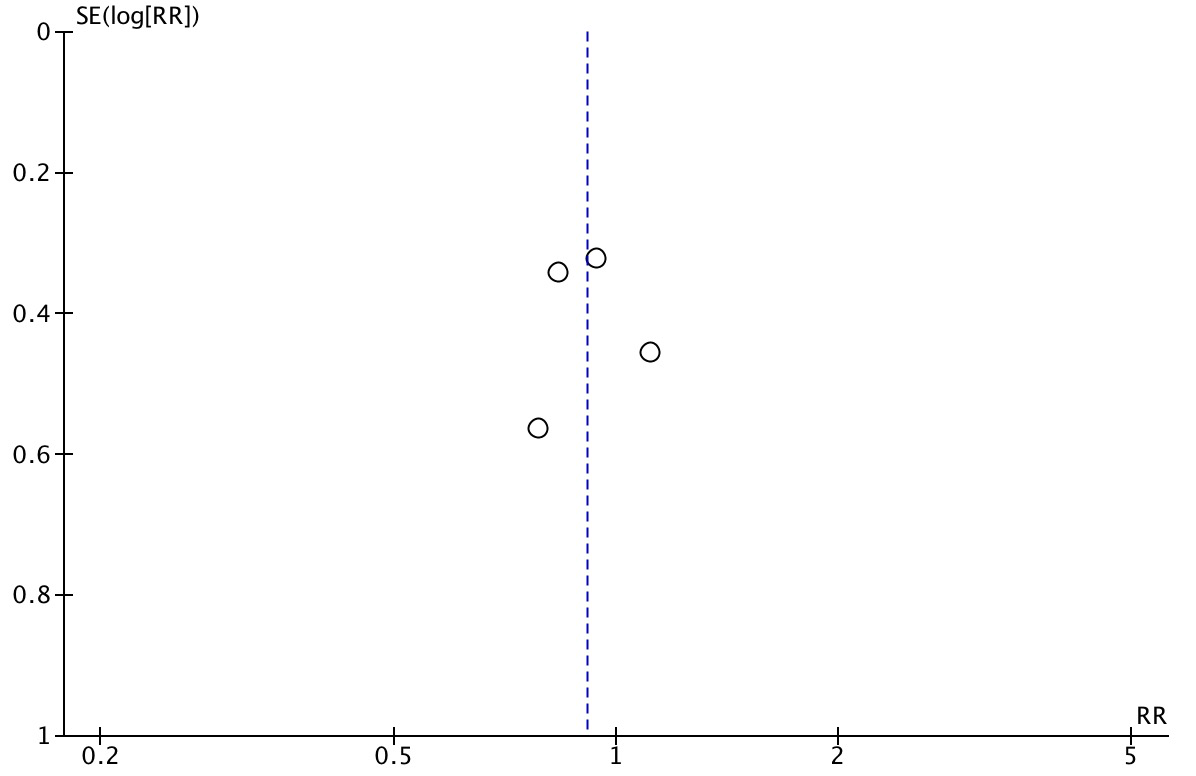


**Supplementary Figure S3**. Funnel plot for cardiovascular mortality.


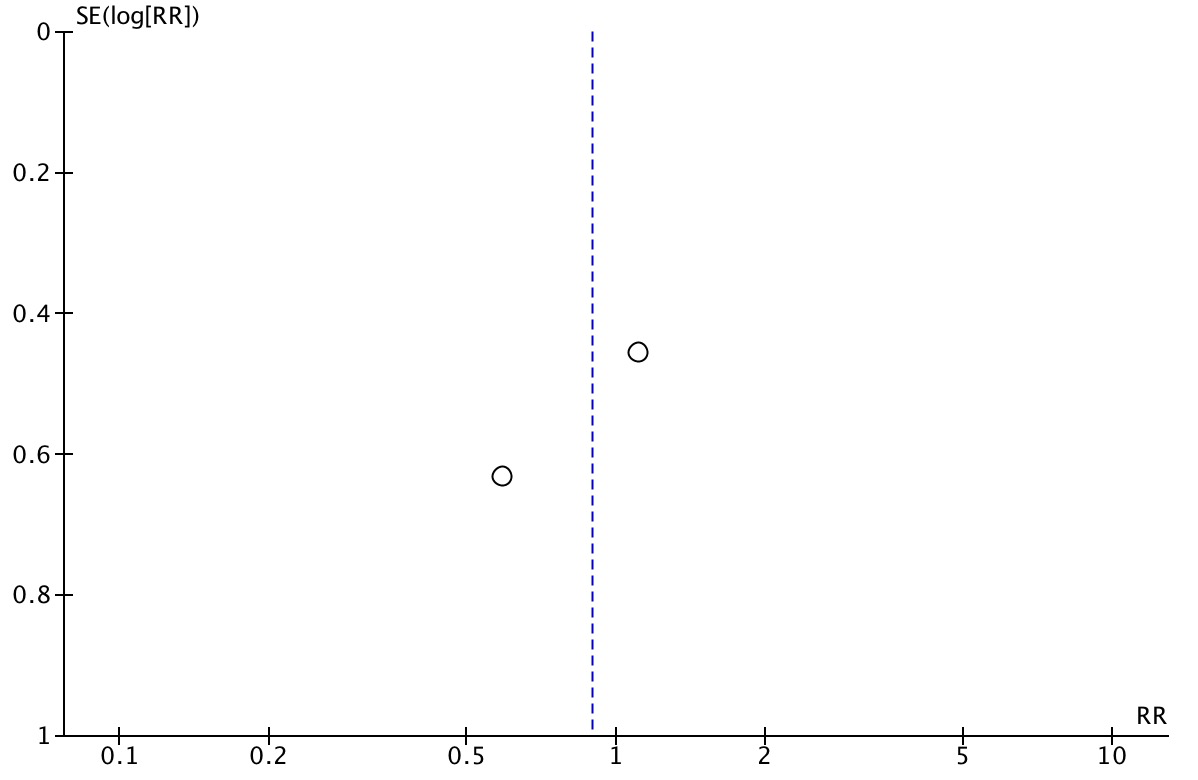


**Supplementary Figure S4**. Funnel plot for stroke.


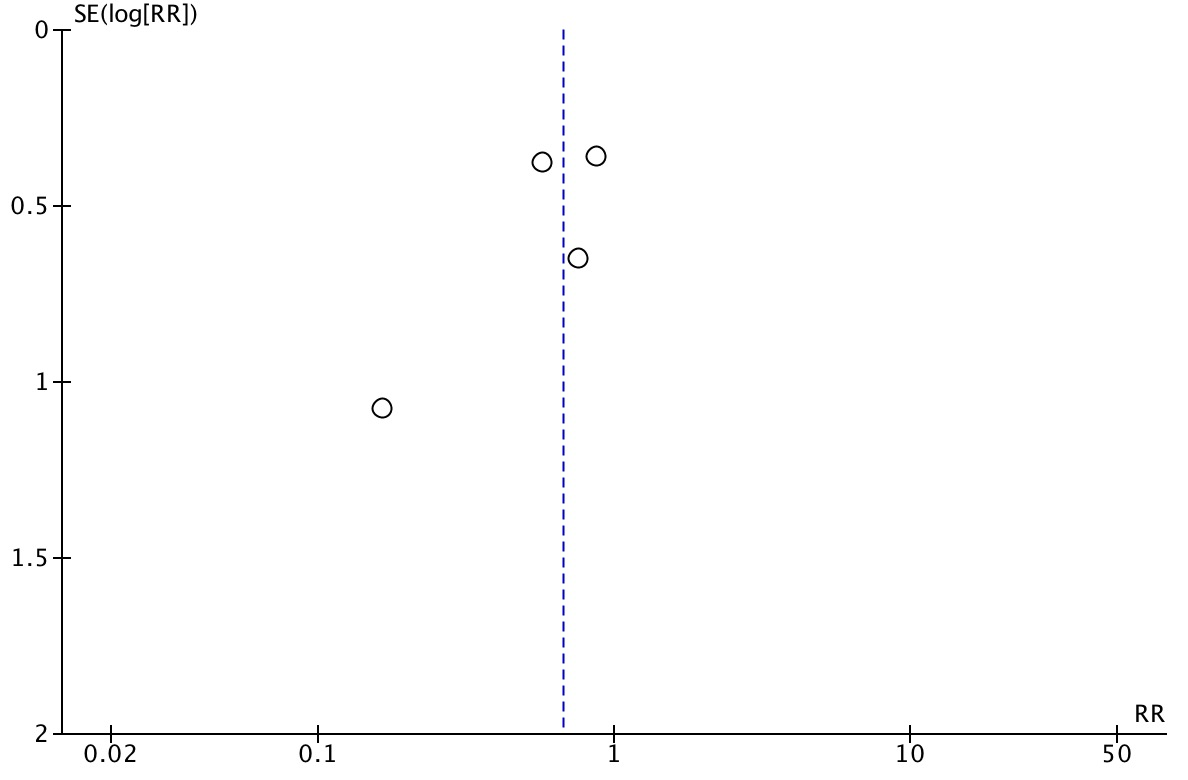


**Supplementary Figure S5**. Funnel plot for closure device failure.


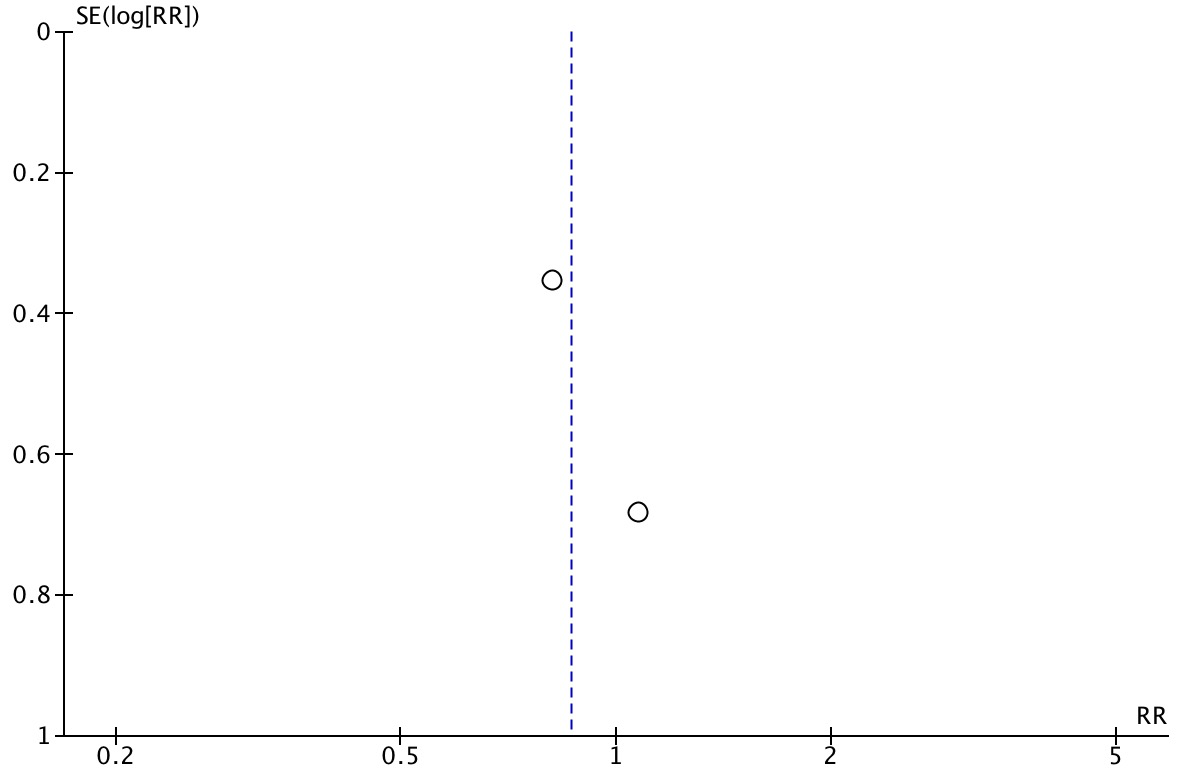


**Supplementary Figure S6**. Funnel plot for major/ life-threatening bleeding


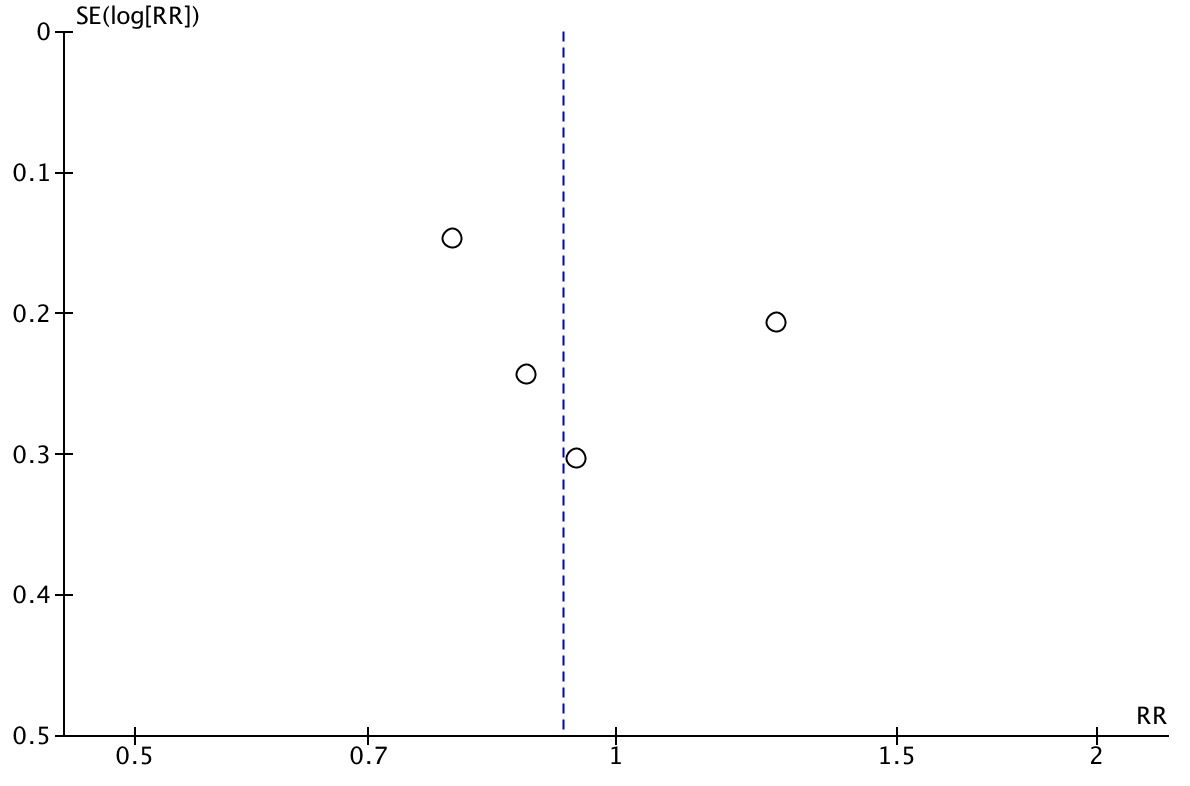


**Supplementary Figure S7**. Funnel plot for major vascular complications


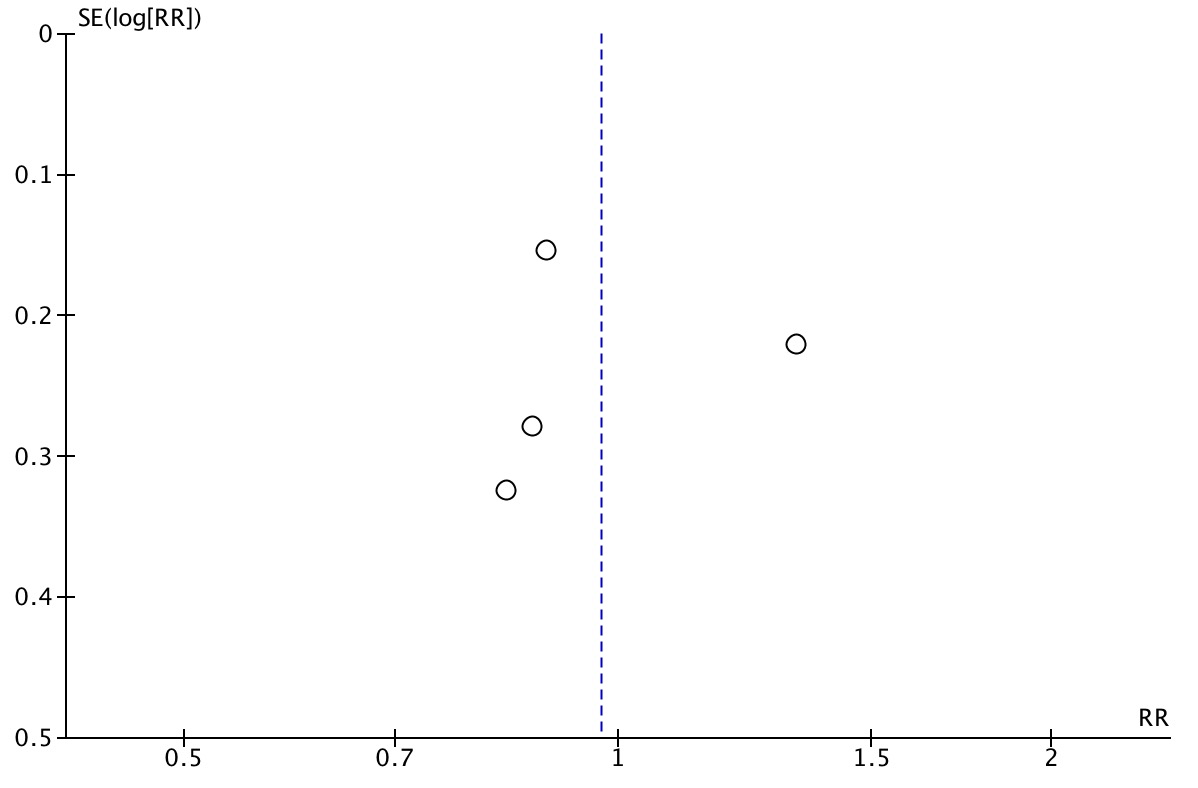

Supplement: Supplementary file 1 [file ajt-33-e31-s001.docx]
